# Nothnagel’s Encyclopedia of Practical Medicine

**Published:** 1904-10

**Authors:** 


NOTHNAGEL'S ENCYCLOPEDIA OF PRACTICAL MEDICINE. American Edition. W. B. Saunders & Co., Philadelphia, Publishers. We have received two large volumes of this series, towit:

TUBERCULOSIS AND ACUTE GENERAL MILIARY TUBERCULOSIS, by Prof. Dr. G. Cornet of Berlin. Edited, with additions, by Walter B. James, M. D., Columbia University, New York. Cloth, $5. 770 pages. And

DISEASES OF THE INTESTINES AND PERITONEUM, by Prof. Dr. Herman Northnagel, Vienna. Edited, with additions, by Humphrey D. Rollerton, M. D., F. R. C. P., Cambridge, Eng. Price, cloth, $5. 1032 pages. Both books being authorized translations from the German, under the editorial supervision of Alfred Stengel, M. D., Professor of Clinical Medicine, University of Pennsylvania.

The great German authority on Tuberculosis gives us an exhaustive work on the subject most opportunely, as public interest is thoroughly aroused to the importance of taking action by governments, to arrest the spread of the devastating scourge, and Tuberculosis Congresses are being held everywhere. Not only medical men, but the people, are aroused, and this splendid work will enlighten them fully on every phase of the subject. We advise every one of our readers to put a copy of it in his library.

The work on Diseases of the Intestines is a whole library within itself, and it elucidates and untangles many of the problems of intestinal troubles that so vex and baffle many practitioners of medicine.

The chapter on Chemic Processes that Occur in the Intestines is by Dr. Fritz Obermeyer. He opens it by saying: "Our knowledge of intestinal digestion is deficient and is not consistent with our knowledge of normal physiology, and still less as regards pathologic perversions. Much less is known about the chemic function of the intestines than about those of the stomach. This is mainly due to the difficulty of obtaining the contents of the intestines (chyme) in a satisfactory state for chemic analysis, but, in addition, the complicated nature of the chemic processes that go on in the intestines makes the solution of the problem far from easy." Dr. Obermeyer then proceeds to give "a condensed summary of the present state of our knowledge of intestinal digestion."

Both these volumes are well printed and gotten out, and easily lead in bringing the reader up to date on the important subjects of which they treat. D.

